# Early Osteogenic and Stromal Marker Responses of Osteoblast-like and Bone-Marrow Stromal Cell Lines to a Hyaluronic Acid-Coated Xenogeneic Bone Graft: An Exploratory In Vitro Analysis

**DOI:** 10.3390/dj14050290

**Published:** 2026-05-12

**Authors:** Yaniv Mayer, Hia Abu Sada, Hadar Zigdon Giladi, Eran Gabay, Ofri Doppelt-Flikshtain, Ofir Ginesin

**Affiliations:** 1 Ruth and Bruce Rappaport, Faculty of Medicine at the Technion, Haifa 3525433, Israel; 2 Department of Periodontology, Rambam Health Care Campus, Haifa 3109601, Israel; 3 Laboratory for Bone Repair, Clinical Research Institute at Rambam (CRIR), Haifa 3109601, Israel

**Keywords:** hyaluronic acid, xenograft, bone regeneration, osteogenesis, deproteinized bovine bone mineral, RUNX2, COL1A1, in vitro

## Abstract

**Background:** To investigate whether coating xenogeneic bone grafts with hyaluronic acid influences early osteogenic and fibrotic marker expression in vitro. **Methods:** Three xenograft materials were evaluated, including one hyaluronic acid-coated product and two uncoated deproteinized bovine bone mineral products, all commercially available. Human osteoblast-like cells (U2OS) and bone marrow stromal cells (HS5) were cultured with material extracts. Proliferation was assessed using XTT assay at 24 and 48 h. Cell adhesion was evaluated through fluorescence microscopy. Osteogenic markers (RUNX2, COL1A1) and fibrotic markers (COL3A1, TGF-β3) were quantified using quantitative real-time PCR. Statistical analysis employed one-way ANOVA with Benjamini–Krieger–Yekutieli (BKY) two-stage FDR correction for datasets that met the normality assumption, and the Kruskal–Wallis test with Dunn’s post hoc test for non-normally distributed data (HS5 XTT assay). Pairwise comparisons were restricted to each xenograft group versus the untreated control; an adjusted *p*-value < 0.05 was considered statistically significant. **Results:** At 48 h, the HA-coated xenograft (Xeno1) showed the highest mean metabolic activity in U2OS cells (0.538 ± 0.056) compared with the uncoated Xeno2 (0.450 ± 0.120) and Xeno3 (0.439 ± 0.073); however, after FDR correction no statistically significant differences were observed between groups. The coated material was associated with upregulation of early osteogenic markers, 2.61-fold RUNX2 upregulation (*p* = 0.01) compared to untreated cells. Both coated and uncoated xenografts demonstrated equivalent suppression of fibrotic markers in HS5 cells, reducing COL3A1 by 92.7% (*p* = 0.001) and TGF-β3 by 92.1% (*p* = 0.001). **Conclusions:** These exploratory in vitro findings suggest that HA coating may enhance early osteogenic marker expression. The observed effects on stromal markers warrant further investigation using primary cells, additional fibrotic endpoints (e.g., TGF-β1, ACTA2), and in vivo models before translational conclusions can be drawn.

## 1. Introduction

Bone grafting is a common and essential surgical procedure in regenerative medicine, used to repair bone defects arising from trauma, disease, or atrophy, and to provide structural support for dental implants [1,2]. As the second most transplanted tissue after blood, the global demand for bone graft materials is exceedingly high [3]. A central challenge in tissue engineering is developing materials that can mimic the complex hierarchical structure and biological properties of natural bone [4]. Current solutions are classified into four main categories: autografts (from the patient), allografts (from a human donor), xenografts (from an animal source), and alloplasts (synthetic) [5]. For years, autografts have been considered the “gold standard” due to their excellent osteogenic, osteoinductive, and osteoconductive properties [6,7]. However, their use is constrained by significant drawbacks, including the need for a second surgical site, potential donor site morbidity, increased operative time, and a limited available quantity [6,7,8].

To overcome these limitations, xenografts, particularly deproteinized bovine bone mineral (DBBM), have become a widespread and popular alternative in dental and cranio-maxillofacial applications [9,10]. These grafts offer high availability, excellent biocompatibility, and a three-dimensional mineral structure that closely resembles human bone, serving as an effective osteoconductive scaffold for the ingrowth of blood vessels and bone-forming cells [9,11,12]. They are routinely used in clinical procedures such as alveolar ridge preservation, sinus augmentation, and guided bone regeneration [13,14,15]. Despite their advantages, the clinical efficacy of xenografts can be limited. They function primarily as passive scaffolds, lacking intrinsic osteoinductive properties, and their slow resorption rate can impede optimal regeneration [16]. Furthermore, variations in processing methods among manufacturers, such as sintering temperatures, profoundly influence the graft’s biological and physical properties, underscoring the need to enhance existing materials [17].

Consequently, there is growing interest in combining xenografts with bioactive agents capable of actively modulating the cellular environment to favor bone formation over fibrotic encapsulation.

Hyaluronic acid (HA), a long, unbranched polysaccharide composed of repeating disaccharides of D-glucuronic acid and N-acetyl-D-glucosamine and a major component of the extracellular matrix, is known to play a crucial role in wound healing, cell migration, and inflammation modulation [18,19]. The biological properties of HA are significantly influenced by its molecular weight; high-molecular-weight (>500 kDa) HA generally exhibits anti-inflammatory and immunosuppressive effects, whereas low-molecular-weight (<500 kDa) fragments tend to be pro-inflammatory and pro-angiogenic [20,21]. Hyaluronic acid (HA) exerts its biological activity primarily through binding to the CD44 cell surface receptor. Through this interaction, HA activates downstream signaling pathways, including Wnt/β-catenin, TGF-β, and various MAPK kinase cascades that regulate key cellular processes such as proliferation, migration, differentiation, and adhesion. This mechanism operates across multiple cell types, including epithelial cells, fibroblasts, mesenchymal stem cells, and endothelial cells, where HA–CD44 interaction governs cytoskeletal remodelling, cell survival, and tissue repair responses [22,23].

To evaluate the biological response, key molecular markers were selected based on their established roles in bone biology. RUNX2 is recognized as the master regulator of osteoblast differentiation and is essential in the early stages of bone formation [24], while Collagen Type I (COL1A1) is the most abundant protein in the bone matrix, providing the scaffold for mineral deposition [25]. Conversely, Collagen Type III (COL3A1) and Transforming Growth Factor-beta 3 (TGF-β3) are key markers relevant to stromal cell responses. COL3A1 is a well-established marker of fibrotic tissue. TGF-β3 was included to capture changes in TGF-β signaling; however, its role in fibrosis is complex, as it has been reported to reduce scar formation in certain tissue contexts [26,27]. The U2OS human osteosarcoma cell line, derived from a tibial osteosarcoma, is frequently employed as an osteoblastic model, while HS5 is a fibroblast-like bone marrow stromal line that closely mimics the characteristics of primary mesenchymal stromal cells [28,29].

The purpose of this study, therefore, is to investigate whether coating a xenogeneic bone graft with hyaluronic acid influences early osteogenic and stromal cell marker expression in an exploratory in vitro setting.

The working hypothesis of this study is that the addition of hyaluronic acid to xenogenic bone grafts will enhance the proliferation and adhesion of osteogenic and stromal cells. We postulate that this will lead to an upregulation of key osteogenic markers (RUNX2 and COL1A1) and altered expression of stromal markers (COL3A1 and TGF-β3) when compared to xenogenic bone grafts without a hyaluronic acid coating.

## 2. Material and Methods

### 2.1. Xenograft Materials

Three commercially available xenograft materials were evaluated. The experimental group (Xeno1) was Cerabone Plus (Botiss Biomaterials GmbH, Zossen, Germany), a bovine-derived bone substitute pre-coated with high-molecular-weight hyaluronic acid. The first control (Xeno2) was Cerabone (Botiss Biomaterials GmbH, Germany), the uncoated counterpart of Cerabone Plus. Because Xeno1 and Xeno2 share the same base material and manufacturer, differing only in the presence of the HA coating, this pair serves as the primary mechanistic comparison for isolating the effect of HA biofunctionalization. The second control (Xeno3) was Bio-Oss (Geistlich Pharma AG, Wolhusen, Switzerland), a widely used deproteinized bovine bone mineral included as an independent commercial benchmark. As Xeno3 differs from the Botiss products in both manufacturer and deproteinization process, any differences involving this material cannot be attributed to the HA coating per se and are interpreted accordingly.

### 2.2. Cell Lines and Culture Conditions

Two human cell lines were selected to model the distinct cellular responses involved in bone regeneration. To evaluate osteogenic potential, the U2OS human osteosarcoma cell line (ATCC, Manassas, VA, USA), a well-established osteoblast-like model, was utilized. To assess fibrotic responses, the HS-5 human bone marrow stromal cell line (ATCC, MD, USA), which exhibits a fibroblast-like phenotype, was chosen. U2OS cells were maintained in low-glucose Dulbecco’s Modified Eagle Medium (DMEM) (Biological Industries Ltd., Beit-Haemek, Israel), while HS-5 cells were maintained in high-glucose DMEM (Biological Industries Ltd., Beit-Haemek, Israel), both supplemented with 10% fetal bovine serum (FBS) (Biological Industries Ltd., Beit-Haemek, Israel) and 1% penicillin/streptomycin (Biological Industries Ltd., Beit-Haemek, Israel). Both cell lines were cultured under standard conditions at 37 °C in a humidified incubator with 5% CO_2_. The culture medium was replenished twice weekly, and cells were subcultured using a 0.05% trypsin-EDTA solution upon reaching 75–80% confluence. To enable fluorescence-based visualization in the adhesion assay, a GFP-expressing U2OS subline was generated via lentiviral transduction as previously described (Doppelt-Flikshtain, 2024) [30]. Briefly, a non-targeting shRNA sequence was cloned into the GIPZ Lentiviral Human shRNA plasmid (RHS4531-EG4318, Horizon Discovery, Lafayette, CO, USA), which co-expresses GFP as a reporter. Lentiviruses were packaged by co-transfection with pMD2.G and psPAX2 (Addgene plasmid #12259 and #12260, respectively) using the calfectin reagent (SL100478, SignaGen Laboratories, Frederick, MD, USA). 293T cells were utilized as packaging cells. U2OS cells were infected with the lentiviruses supplemented with 8 μg/mL polybrene (TR-1003-G, Sigma-Aldrich, St. Louis, MO, USA) and selected in the presence of 5 μg/mL puromycin (J67236, Alfa Aesar, Ward Hill, MA, USA). Transduction efficiency was evaluated using real-time PCR and successful GFP expression was confirmed via fluorescence microscopy. The U2OS cell line was selected as an osteoblast-like screening model because of its well-characterized expression of osteogenic markers and its extensive use in biomaterial evaluation studies. The HS-5 cell line was chosen for its fibroblast-like phenotype and its responsiveness to substrate properties. While neither cell line fully recapitulates primary cell physiology, both are established models for initial in vitro screening.

### 2.3. Proliferation Assay

Cellular metabolic activity, used as an indirect indicator of viability and proliferative capacity, was assessed using a colorimetric XTT Cell Proliferation Kit (Biological Industries Ltd., Beit-Haemek, Israel). Each xenograft sample (0.25 g) was immersed in the appropriate DMEM medium and incubated for 24 h at 37 °C with 5% CO_2_ to produce conditioned extracts. Importantly, the Xeno1 (HA-coated) extract was diluted 2-fold with fresh DMEM prior to application to cells, whereas the Xeno2 and Xeno3 extracts were applied without dilution. This concentration difference should be considered when interpreting comparisons involving Xeno1, as it represents a potential confound that limits attribution of observed differences solely to the HA coating.

U2OS cells (6000 cells/well) and HS5 cells (10,000 cells/well) were seeded in 96-well plates and incubated with the conditioned extracts for 24 and 48 h under standard conditions (37 °C, 5% CO_2_). Metabolic activity was quantified by measuring absorbance at 475 nm, with the non-specific reference absorbance at 660 nm subtracted to correct for background. Blank values (medium only, without cells, prepared in quadruplicate) were subtracted from all experimental readings prior to analysis.

Cells cultured in their respective standard media without xenograft extracts served as untreated controls. Each experiment was performed in three independent biological replicates per group, except for the HS5 XTT Xeno3 group at 48 h, for which two biological replicates were available; this is noted as a limitation. In cases where a clear technical error was identified, the specific reading was categorized as a technical exclusion and omitted from the dataset prior to analysis.

### 2.4. Adhesion Assay

Cellular adhesion to the xenograft surfaces was evaluated using fluorescence microscopy. Xenograft granules (0.06 g, 0.5–1 mm particle size) were incubated with 250,000 cells in 6-well plates for 24 and 48 h. Following incubation, the cells were fixed with 4% formaldehyde, permeabilized with 0.5% Triton X-100, and stained with DAPI to visualize nuclei. The U2OS cells used in this assay expressed GFP following lentiviral transduction (see Section 2.2), allowing for their visualization without additional staining. Images were captured using time-lapse confocal microscopy (Spinning Disk Confocal Olympus IXplore-Spin, Tokyo, Japan) at 5× and 10× magnification to observe cell distribution and morphology on the material surfaces.

### 2.5. Gene Expression Analysis

Quantitative real-time PCR (qRT-PCR) was employed to analyze the expression of key genes indicative of osteogenic and fibrotic pathways. Total RNA was isolated from 5 × 10^5^ cells using the E.Z.N.A.^®^ HP Total RNA Kit (Omega BIO-TEK, Norcross, GA, USA), and one microgram of RNA was subsequently reverse-transcribed into cDNA. cDNA synthesis was performed using the qScript^®^ cDNA Synthesis Kit (1 µg RNA), with primer annealing at 22 °C for 5 min, extension at 42 °C for 30 min, and enzyme inactivation at 85 °C for 5 min. qRT-PCR was performed in three independent biological replicates for *RUNX2*, *COL3A1*, and *TGF-β3*, and in four independent biological replicates for *COL1A1*, with each biological replicate run in technical triplicate using SYBR Green (Fast SYBR™ Green Master Mix, Applied Biosystems, Foster City, CA, USA). Melt curve analysis was performed after each qRT-PCR run to confirm amplification specificity. For the U2OS cells, the expression of osteogenic markers *RUNX2* (Primer sequence: Forward (F) 5′-TAAGAAGAGCCAGGCAGGTG-3′ Reverse (R) -5′-TGGCAGGTACGTGTGGTAGT-3′) and *COL1A1* (F: 5′-GCCAAGACGAAGACATCCCA-3′, R: 5′-GCACCATCATTTCCACGAGC-3′) was quantified. For the HS5 cells, the expression of fibrotic markers *COL3A1* (F: 5′-AAGGAAATGATGGTGCTCCTG-3′, R: 5′-AGCCTTGTAATCCTTGTGGAC-3′) and *TGF-β3* (F-5′ GAGCTCTTCCAGATCCTTCG -3′, R: 5′-TTTCTAGACCTAAGTTGGACTCTC-3′) was assessed. The housekeeping gene *HPRT* (5′-GACCAGTCAACAGGGGACAT-3′, R: 5′-CCTGACCAAGGAAAGCAAAG-3′) was used for normalization, and relative gene expression was calculated as fold change using the 2^−ΔΔCt^ method [31]. For statistical inference, analyses were performed on the normalized ΔCt values to ensure a more appropriate distribution for parametric testing.

### 2.6. Statistical Analysis

All quantitative data were analysed in GraphPad Prism 9.0. For each assay and group, normality was first assessed using the Shapiro–Wilk test. Datasets that met the normality assumption (U2OS XTT at 24 h and 48 h, and qRT-PCR data for RUNX2, COL1A1, COL3A1, and TGF-β3) were analysed using one-way ANOVA. Pairwise comparisons were restricted to each xenograft group (Xeno1, Xeno2, Xeno3) versus the untreated control; we did not perform all pairwise contrasts between xenograft groups as a primary analysis. Within each assay, the family of treatment-versus-control contrasts was corrected for multiple comparisons using the Benjamini–Krieger–Yekutieli (BKY) two-stage linear step-up procedure at a false-discovery rate of q = 0.05. Data that did not meet the normality assumption (HS5 XTT) were analysed using the Kruskal–Wallis test, followed by Dunn’s multiple-comparisons test with Bonferroni-type adjustment of *p*-values as implemented in GraphPad Prism. Unpaired Student’s *t*-tests were not used in the final analyses; all group comparisons employed ANOVA (parametric) or Kruskal–Wallis (non-parametric) as described. The ROUT method (Q = 1%) was applied to qRT-PCR data and identified no outliers; for the HS5 XTT assay, one observation in the Xeno1 group at 24 h was excluded prior to analysis as a pre-specified technical exclusion (handling error), not as a statistical outlier. All *p*-values and significance indicators shown in figures are multiplicity-adjusted (q-values after BKY for ANOVA-based datasets; Dunn-adjusted *p*-values for HS5 XTT); raw, uncorrected *p*-values are not displayed in the figures. Biological replicates per assay and group were: U2OS XTT, *n* = 3 per group; HS5 XTT, *n* = 3 per group except Xeno3 at 48 h (*n* = 2), a limitation acknowledged in the Results and Discussion; U2OS qRT-PCR RUNX2, *n* = 3; U2OS qRT-PCR COL1A1, *n* = 4; HS5 qRT-PCR COL3A1 and TGF-β3, *n* = 3; and adhesion assay, *n* = 3 qualitative replicates per group. Each biological replicate was run in technical triplicate for qRT-PCR and at least in technical duplicate for XTT. All results are expressed as mean ± standard deviation (SD), and an adjusted *p*-value < 0.05 was considered statistically significant.

## 3. Results

### 3.1. Metabolic Activity of U2OS Osteoblast-like Cells Exposed to Xenograft Extracts

**Figure 1:**
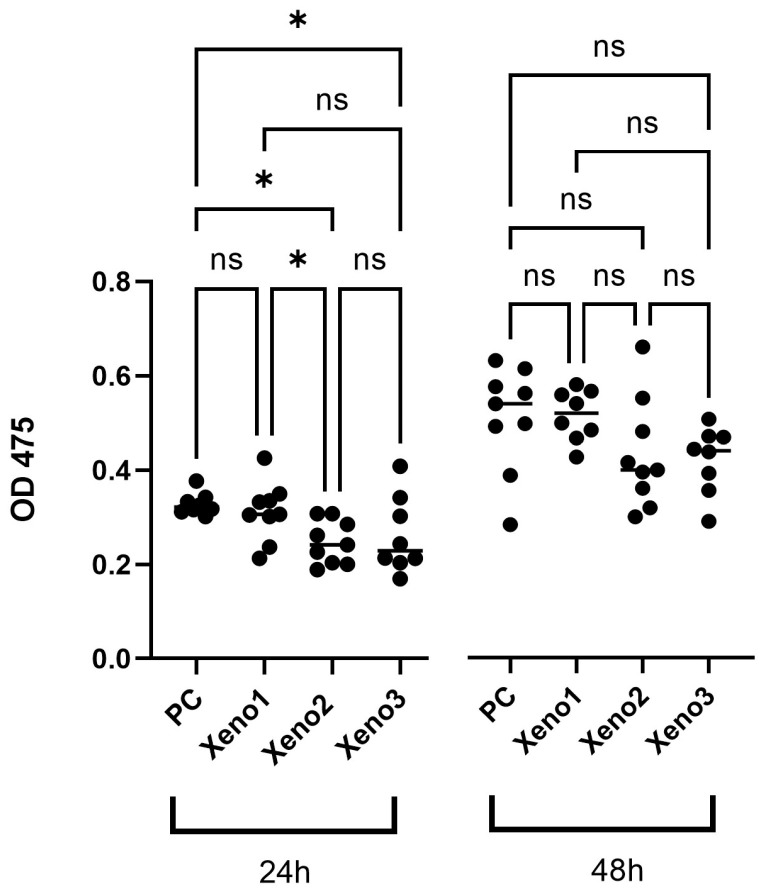
The HA-coated xenograft (Xeno1) showed the highest mean metabolic activity at 48 h compared with the uncoated xenografts; after FDR correction, differences between groups were not statistically significant (*n* = 3 biological replicates). Data are presented as mean ± SD. All *p*-values and significance indicators shown are multiplicity-adjusted (BKY FDR-corrected q-values for ANOVA-based datasets; Dunn-adjusted *p*-values for the HS5 XTT dataset). Raw *p*-values are not displayed. ns: no significant difference (*p* > 0.05) *: *p* < 0.05.

The metabolic activity of U2OS osteoblast-like cells was measured as an indirect indicator of viability and proliferation. At 24 h, mean absorbance values were comparable between groups (Untreated 0.328 ± 0.023; Xeno1 0.312 ± 0.062; Xeno2 0.247 ± 0.046; Xeno3 0.262 ± 0.081; *n* = 3 per group). At 48 h, the HA-coated Xeno1 group showed the numerically highest mean absorbance (0.538 ± 0.056), with Xeno2 (0.450 ± 0.120) and Xeno3 (0.439 ± 0.073) showing lower mean values. After one-way ANOVA with BKY two-stage FDR correction, no statistically significant differences were detected between any xenograft group and the untreated control, or between xenograft groups, at either 24 h or 48 h (Table 1, Figure 1); raw absorbance values for individual replicates are provided in Table S1, Supplementary Materials. This dataset is therefore presented descriptively only. Two caveats are relevant to its interpretation: (i) the Xeno1 extract was applied at a 2-fold dilution relative to Xeno2 and Xeno3 (see Section 2.3), so any numerical differences involving Xeno1 cannot be attributed to the HA coating alone; and (ii) the sample size (*n* = 3 per group) limits the power to detect modest between-group differences.

### 3.2. HA-Coated Xenograft Is Associated with Upregulation of Early Osteogenic Markers

**Figure 2:**
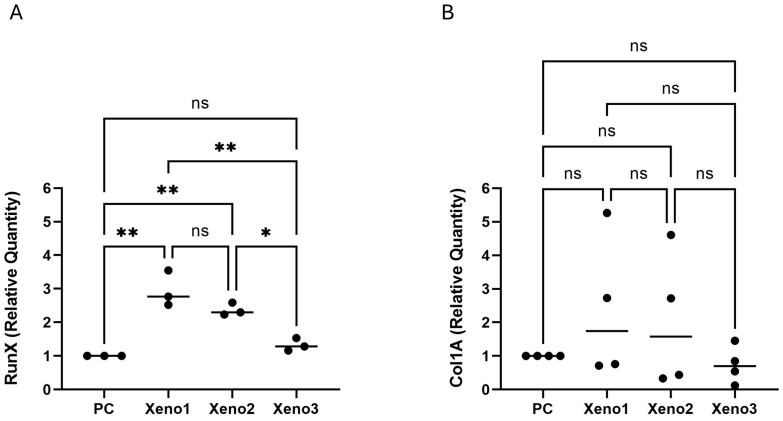
Osteogenic Marker Expression in U2OS Cells. Fold change in (**A**) *RUNX2* (*n* = 3) and (**B**) *COL1A1* (*n* = 4) mRNA expression at 48 h relative to untreated controls, analyzed by one-way ANOVA with BKY FDR correction (q = 0.05). Significance indicators refer to comparisons versus the untreated control (* *p* < 0.05, ** *p* < 0.01, ns = not significant). Data are mean ± SD. As only two transcripts were measured at a single early time point, these findings describe early osteogenic marker expression and do not establish osteogenic differentiation. All *p*-values and significance indicators shown are multiplicity-adjusted (BKY FDR-corrected q-values for ANOVA-based datasets; Dunn-adjusted *p*-values for the HS5 XTT dataset). Raw *p*-values are not displayed.

mRNA expression analysis via qRT-PCR revealed that the HA-coated xenograft induced the strongest osteogenic commitment in U2OS cells. The expression of *RUNX2*, the master regulator of osteoblast differentiation, was upregulated by 2.61-fold change (*p* < 0.01) in the HA-coated group compared to untreated cells. This was substantially higher than the upregulation observed in the uncoated group (Figure 2); raw Ct values for all biological replicates are provided in Tables S3–S6, Supplementary Materials. These results suggest that the HA coating may be associated with early osteogenic transcriptional activity; however, two markers at a single time point are insufficient to establish osteogenic differentiation, which requires confirmation with additional endpoints such as ALP activity, later-stage markers (e.g., SP7/osterix, BGLAP/osteocalcin), mineralization assays, and protein-level validation.

### 3.3. Adhesion of Osteoblast-like Cells

**Figure 3:**
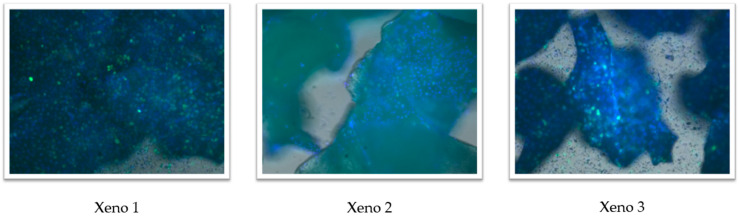
Fluorescence microscopy images of GFP-expressing U2OS cells (green) adhering to the surfaces of the HA-coated xenograft (Xeno1), uncoated Cerabone (Xeno2), and Bio-Oss (Xeno3) after 48 h of incubation. Nuclei are counterstained with DAPI (blue). Images were captured using confocal microscopy (IXplore-Spin, Olympus, Tokyo, Japan) at 10× magnification. The adhesion assay is qualitative; no quantification of adherent cell number or covered area was performed. *n* = 3 independent replicates per group.

Fluorescence microscopy images showed GFP-positive U2OS cells together with DAPI-stained nuclei in proximity to particles of all three xenograft materials after 48 h of incubation (Figure 3). Because the assay was qualitative, with no quantification of adherent cell number or of covered area, and because scale bars were not included on the images, no comparative or mechanistic conclusions regarding cell adhesion are drawn from these observations. The images are presented for descriptive visualisation only.

### 3.4. Xenografts Are Associated with Reduced Expression of Stromal Markers COL3A1 and TGF-β3

**Figure 4:**
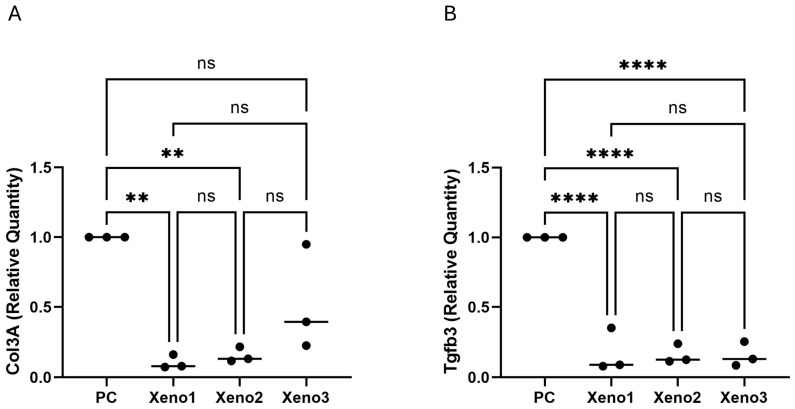
Stromal Marker Gene Expression in HS5 Cells. Fold change in (**A**) *COL3A1* and (**B**) *TGF-β3* expression after 48 h of exposure to xenograft extracts. All xenograft groups were associated with a significant reduction in the expression of both transcripts relative to the untreated control, with no significant differences between xenograft groups (** *p* < 0.01, **** *p* < 0.0001, ns = no significant difference; *n* = 3 biological replicates). These findings describe altered stromal marker expression and should not be interpreted as mechanistic evidence of an anti-fibrotic phenotype. All *p*-values and significance indicators shown are multiplicity-adjusted (BKY FDR-corrected q-values for ANOVA-based datasets; Dunn-adjusted *p*-values for the HS5 XTT dataset). Raw *p*-values are not displayed.

In HS5 bone-marrow stromal cells, exposure to extracts from all three xenograft materials was associated with a marked reduction in COL3A1 and TGF-β3 transcript levels relative to the untreated control (approximately 92% reduction for each transcript; adjusted *p* < 0.001; *n* = 3 biological replicates; Figure 4). There were no statistically significant differences between the three xenograft groups. Because only two transcripts were measured at a single early time point, in an immortalised cell line, and without protein-level validation, these observations are reported strictly as changes in stromal-marker transcript expression and should not be interpreted as evidence of an anti-fibrotic phenotype. Canonical fibrosis endpoints such as ACTA2/α-SMA, CTGF, fibronectin, and extracellular-matrix deposition were not assessed in this study and would be required to make such an interpretation.

### 3.5. Stromal Cell Proliferation

The metabolic activity of HS5 stromal cells showed a different pattern compared to U2OS cells. While all groups recovered and proliferated between 24 and 48 h, no statistically significant differences were observed between any of the xenograft groups at the 48 h time point (Table 2); raw absorbance values for individual replicates are provided in Table S2, Supplementary Materials. One well in the Xeno1 group at the 24 h timepoint was excluded before analysis as a pre-specified technical exclusion (handling error), in accordance with Section 2.3; this observation was not flagged as a statistical outlier by ROUT.

**Table 2:** HS5 Cell Line Metabolic Activity. Metabolic activity (absorbance at 475 nm) of HS5 cells after 24 and 48 h.

Treatment Group	24 Hours	48 Hours	Biological Replicates
Untreated Control (HS5)	0.406 ± 0.069	0.546 ± 0.079	3
Xeno1	0.317 ± 0.030	0.478 ± 0.119	3
Xeno2	0.316 ± 0.039	0.465 ± 0.082	3
Xeno3	0.379 ± 0.11	0.513 ± 0.179	2

Note: For Xeno3 at 48 h, *n* = 2 biological replicates were available; this uneven replication is acknowledged as a limitation of the HS5 XTT experiment.

## 4. Discussion

This exploratory in vitro study examined whether coating a xenogeneic bone graft with hyaluronic acid (HA) is associated with changes in early osteogenic and stromal marker expression. The primary comparison of interest was between the HA-coated Xeno1 and its uncoated counterpart Xeno2, which share the same base material and manufacturer. Within this pair, the numerical trend in U2OS metabolic activity did not reach statistical significance after FDR correction and should therefore be interpreted as descriptive only. Under the tested conditions, exposure to extracts of the HA-coated commercial preparation was associated with upregulation of the early osteogenic transcripts RUNX2 and COL1A1 in U2OS cells, while exposure to all three xenograft preparations was associated with a reduction in COL3A1 and TGF-β3 transcript levels in HS5 stromal cells. We note that the Xeno1 extract was applied at a 2-fold dilution relative to Xeno2 and Xeno3, and that no independent physicochemical characterisation of the materials was performed; accordingly, differences involving Xeno1 are described as observations associated with the tested HA-coated preparation under the reported conditions, rather than as effects attributable to HA coating per se. These observations are compatible with previous reports that HA biofunctionalisation of bone substitutes can modulate osteogenic-lineage responses and with the known bioactive role of HA in bone healing [24,32,33]. However, because the Xeno1 extract was diluted relative to the comparators, no independent physicochemical characterisation was performed, and the materials differ in manufacturer and deproteinisation process, we do not invoke specific mechanisms, such as HA–CD44 signalling or molecular-weight-dependent effects of HA on fibroblast activation [20,34,35] to explain the present findings. Such mechanisms remain plausible but would require direct testing with molecular-weight-defined HA, receptor blockade, and primary cells. Calcium release from xenografts can activate calcium-sensing receptors on osteoprogenitor cells, leading to increased RUNX2 expression through MAPK-dependent mechanisms. The different calcium release profiles of the tested materials, influenced by their manufacturing processes, may therefore contribute to the observed variations in RUNX2 expression between groups. The anti-fibrotic properties observed are particularly significant and are consistent with the known biology of high molecular weight (HMW) HA. HMW HA (typically > 1000 kDa) exhibits anti-inflammatory and immunosuppressive effects, while low-molecular-weight (LMW) fragments (<200 kDa) produced during tissue injury tend to promote inflammation and fibroblast activation [20]. This molecular-weight-dependent duality extends to fibrocyte differentiation: HMW HA potentiates fibrocyte differentiation from monocytes, whereas LMW HA inhibits this process through differential effects on CD44 receptor expression. In the context of wound healing, HMW HA promotes fibroblast migration and maintains a moist microenvironment conducive to healing while potentially limiting excessive scar formation [34,35]. The downregulation of *TGF-β3* observed in the present study may therefore reflect HA’s interaction with the CD44 receptor, which can interfere with fibrotic signaling cascades and modulate the broader wound healing response [20].

The differential responses between the U2OS osteoblast-like cells and the HS5 stromal cells highlight the importance of cell type selection in biomaterial evaluation, as different bone-derived cells can exhibit unique reactions [36]. Bio-Oss is processed at lower temperatures (~300 °C), retaining more organic components, while Cerabone products undergo high-temperature sintering (>1200 °C). These residual organic components may provide biological cues that influence cellular proliferation, particularly for bone marrow-derived cells responsive to growth factors and matrix proteins [37,38]. In the U2OS osteoblast-like model, exposure to the HA-coated preparation was associated with upregulation of early osteogenic transcripts; because U2OS is a cancer-derived line, this observation would need to be confirmed in primary osteoblasts and with additional endpoints (ALP activity, later-stage markers such as SP7/osterix and BGLAP/osteocalcin, mineralisation, and protein-level validation) before any statement about osteogenic differentiation can be made. [39]. The HS5 cells, representing bone marrow stromal cells, served as a more discerning model, which aligns with research showing that mesenchymal stromal cells are highly responsive to substrate properties and can be sensitive indicators of biocompatibility [40]. This model proved informative in revealing consistent changes in stromal marker expression, although the present data do not establish a bona fide anti-fibrotic effect and should be interpreted accordingly.

While these results are suggestive of a biological effect of HA coating, the exploratory nature of the study, including the use of immortalized cell lines, a limited marker panel, a single early time point, and qualitative adhesion data precludes strong translational claims. In vivo studies and primary cell validation are essential before clinical implications can be drawn.

However, this study has several limitations. The use of immortalized cell lines in a two-dimensional in vitro model does not fully replicate the complex cellular and molecular interactions of the in vivo environment, where multiple cell types, growth factors, and mechanical stimuli influence the regenerative process. The relatively short duration of the experiment captures only the initial cellular responses and may not reflect long-term tissue maturation and remodeling. Furthermore, no independent physicochemical characterization (e.g., SEM, FTIR, XPS, or quantification of surface-bound HA) was performed. Consequently, the observed biological responses cannot be attributed solely to HA coating, as differences in sintering temperature, surface chemistry, or microtopography between the commercial products may also contribute. In addition, replication was also uneven, notably the HS5 XTT Xeno3 group at 48 h (*n* = 2), limiting statistical power to detect small differences. Most importantly, the two-fold dilution of the Xeno1 extract relative to Xeno2 and Xeno3 inherently limits mechanistic attribution of the observed effects to the HA coating itself; future comparisons should use matched extract concentrations to isolate the coating’s effect. We also acknowledge the absence of scale bars on the confocal microscopy images as a limitation of the adhesion assay reporting.

Future research should aim to validate these promising findings using more physiologically relevant models, such as co-culture systems with primary human osteoblasts and fibroblasts or 3D culture models. Ultimately, in vivo studies in animal models, combined with expanded fibrosis marker panels and protein-level endpoints, are warranted to determine whether the observed changes in stromal marker expression translate into a genuine anti-fibrotic effect relevant to clinical bone regeneration.

## 5. Conclusions

In this exploratory in vitro study using immortalised cell lines and a limited marker panel at a single early time point, exposure to extracts of an HA-coated xenogeneic bone graft (Xeno1) was associated with numerically higher but not statistically significant metabolic activity in U2OS osteoblast-like cells, and with upregulation of the early osteogenic transcripts RUNX2 and COL1A1 relative to the untreated control. In HS5 bone-marrow stromal cells, exposure to extracts of all three xenograft materials was associated with a reduction in COL3A1 and TGF-β3 transcript levels, with no significant differences between the three xenograft groups. These findings should be considered hypothesis-generating and require validation using primary cells, expanded marker panels, protein-level confirmation, and in vivo models before clinical relevance can be established.

## Figures and Tables

**Table 1:** U2OS Cell Line Metabolic Activity. Metabolic activity (absorbance at 475 nm) of U2OS cells after 24 and 48 h of exposure to xenograft extracts. Data are shown as mean ± SD.

Treatment Group	24 Hours	48 Hours	Biological Replicates
Untreated Control (U2OS)	0.328 ± 0.023	0.532 ± 0.117	3
Xeno1	0.312 ± 0.062	0.538 ± 0.056	3
Xeno2	0.247 ± 0.046	0.450 ± 0.120	3
Xeno3	0.262 ± 0.081	0.439 ± 0.073	3

## Data Availability

All raw datasets supporting the findings of this manuscript are provided as Supplementary Material accompanying this submission. Additional details are available from the corresponding author upon reasonable request.

## Supplementary Materials

The following supporting information can be downloaded at https://www.mdpi.com/article/10.3390/dj14050290/s1: Table S1: U2OS XTT proliferation assay. Raw absorbance values (475 nm, with 660 nm reference subtracted) recorded at 24 h and 48 h for the untreated control and for the three xenograft-extract groups (Xeno1, Xeno2, Xeno3) across biological replicates. Table S2: HS5 XTT proliferation assay. Raw absorbance values (475 nm, with 660 nm reference subtracted) recorded at 24 h and 48 h for the untreated control and for the three xenograft-extract groups (Xeno1, Xeno2, Xeno3) across biological replicates. Table S3: qRT-PCR raw Ct values, biological replicate 1. HPRT (housekeeping gene) and COL1A1 measurements in U2OS cells exposed to the untreated control and to the three xenograft extracts, with per-sample average Ct, standard deviation, ΔCt, ΔΔCt, and relative quantification (RQ). Table S4: qRT-PCR raw Ct values, biological replicate 2. HPRT (housekeeping gene), RUNX2, and COL1A1 measurements in U2OS cells exposed to the untreated control and to the three xenograft extracts, with per-sample average Ct, standard deviation, ΔCt, ΔΔCt, and RQ. Table S5: qRT-PCR raw Ct values, biological replicate 3. HPRT (housekeeping gene), RUNX2, and COL1A1 measurements in U2OS cells across the same four conditions, with per-sample average Ct, standard deviation, ΔCt, ΔΔCt, and RQ. Table S6: qRT-PCR raw Ct values, biological replicate 4. HPRT (housekeeping gene), RUNX2, and COL1A1 measurements in U2OS cells across the same four conditions, with per-sample average Ct, standard deviation, ΔCt, ΔΔCt, and RQ. No additional references are cited within the Supplementary Materials.
